# Sternal Closure Techniques Using Steel Wires and Predictors of Sternal Wound Complications: A Randomized Controlled Trial

**DOI:** 10.21470/1678-9741-2026-0056

**Published:** 2026-06-12

**Authors:** Mohamed A. Mogahed, Mohamed M. Abdelaal, Wael M. Elfeky, Ahmed R. Elghanam, Mohamed M. Elesawy, Mohamed M. Abdelalim

**Affiliations:** 1 Department of Cardiothoracic Surgery, Faculty of Medicine, Kafr Elsheikh University, Kafr El Sheikh, Egypt

**Keywords:** Sternal Closure, Sternal Wound Complications, Cardiac Surgery, Sternal Dehiscence

## Abstract

**Introduction:**

Median sternotomy is the gold standard for cardiac surgery but carries a significant risk of wound complications, including sternal dehiscence and wound infections.

**Objective:**

This study aimed to compare the early efficacy and complication rates of three distinct steel-wire sternal closure techniques and identify potential risk factors of complications.

**Methods:**

We conducted a randomized controlled study on patients undergoing cardiac surgery. Patients were randomly allocated into three groups of sternal closure techniques: simple interrupted (Group A), figure-of-eight (Group B), or a modified combined technique (Group C). Baseline characteristics, intraoperative parameters, postoperative recovery outcomes, and sternal wound complications including sternal dehiscence and superficial and deep wound infections were evaluated. Binomial logistic regression was performed to determine independent risk factors for complications.

**Results:**

One hundred sixty-five adult cardiac patients were finally included in the study. All three intervention groups (n = 55) were well-matched regarding baseline characteristics and intraoperative parameters. The incidence of sternal dehiscence (6.1%) and superficial (7.9%) and deep wound infections (3.6%) did not differ significantly among the three wire closure techniques ( *P* > 0.05). However, logistic regression identified numerous factors associated with superficial sternal wound infections including old age, obesity (body mass index > 30), comorbidities, elevated C-reactive protein and HbA1C, prolonged cardiopulmonary bypass time, extended operative time, and longer intensive care unit stay ( *P* < 0.05 for all).

**Conclusion:**

The three steel-wire closure techniques demonstrated comparable early postoperative stability and similar rates of sternal wound complications. Technique choice may be based on surgeon preference.

## INTRODUCTION

**Table:** 

Abbreviations, Acronyms & Symbols				
ASD	= Atrial septal defect		HTN	= Hypertension
AVR	= Aortic valve replacement		ICU	= Intensive care unit
BMI	= Body mass index		LIMA	= Left internal mammary artery
CABG	= Coronary artery bypass grafting		MV	= Mechanical ventilation
CI	= Confidence interval		MVR	= Mitral valve replacement
CKD	= Chronic kidney disease		NYHA	= New York Heart Association
COPD	= Chronic obstructive pulmonary disease		ORs	= Odds ratios
CPB	= Cardiopulmonary bypass		RCT	= Randomized controlled trial
CRP	= C-reactive protein		SAM	= Subaortic membrane
DM	= Diabetes mellitus		SSWI	= Superficial sternal wound infection
DSWI	= Deep sternal wound infection		SWCs	= Sternal wound complications
DVR	= Double valve replacement		TLC	= Total leukocyte count
EF	= Ejection fraction		TVR	= Tricuspid valve replacement
Hb	= Hemoglobin		VIS	= Vasoactive inotropic score

Median sternotomy remains the gold standard incision for accessing the heart and great vessels in adult cardiac surgery, offering unparalleled exposure for the majority of cardiac procedures^[ 1 ]^. This approach was initially described by Milton in 1897 and later popularized for open-heart operations by Julian et al. in 1957^[ 2 ]^. However, sternal wound complications (SWCs), including dehiscence and infection, represent serious postoperative challenges, with reported incidence rates ranging from 0.5% to 6.1%^[ 3 ]^.

Sternal dehiscence, in particular, is a devastating complication, as instability interferes with bone healing, often progressing to deep sternal wound infection (DSWI) or mediastinitis. The associated mortality of mediastinitis remains notably high, estimated between 14% and 47%^[ 4 ]^. Consequently, an ideal sternal closure technique must guarantee mechanical stability, minimize postoperative complications, facilitate short hospitalization, and remain cost-effective^[ 5 ]^.

Despite the existence of more than three dozen recognized sternal closure techniques, many of which claiming superior stability^[ 6 ]^, no single method has yet been established as the definitive gold standard to uniformly prevent complications^[ 7 ]^. The most widely accepted and primary established closure method remains fixation using stainless-steel wires, typically applied using either the simple interrupted or figure-of-eight techniques. This prevalence is attributed to the wires' low cost, ease of use, speed of application, and generally acceptable complication rates^[ 8 ]^.

The choice between simple interrupted and figure-of-eight steel-wire suturing has been the subject of long-standing clinical and biomechanical debate. Some studies have reported high rates of sternal dehiscence using the figure-of-eight technique, such as 9.33% and 12%^[ 9 ]^. Conversely, the simple interrupted technique has also shown widely varying results, from a low dehiscence rate of 1.35% in one series to a rate as high as 12% in another^[ 10 - 12 ]^. Given this persistent lack of consensus, coupled with the absence of localized data from our specific patient population, which presents a distinct profile of risk factors compared to previously studied cohorts, further investigation is warranted.

This randomized controlled trial (RCT) aimed to compare the early postoperative course of three distinct steel-wire sternal closure techniques (simple interrupted, figure-of-eight, and a modified combined technique) and, critically, to identify significant patient- and procedure-related risk factors associated with sternal dehiscence and wound infection in adult cardiac surgery patients.

## METHODS

### Study Design and Approval

This RCT was conducted on adult patients who were scheduled for elective cardiac surgeries at the Department of Cardiothoracic Surgery, Kafrelsheikh University Hospital, Faculty of Medicine, Kafrelsheikh University, from May 2024 to April 2025. The trial adhered strictly to the principles outlined in the Declaration of Helsinki and received ethical approval from The Scientific Research Ethics Committee of Kafrelsheikh University (Approval No. KFSIRB200-219). We have registered the study in the database of the Pan African Clinical Trial Registry (PACTR202512586164819). A written informed consent was obtained from all participating patients prior to their inclusion in the study.

### Eligibility Criteria

The study included adult patients undergoing elective cardiac surgeries requiring a median sternotomy approach. Patients were excluded if they had a history of previous radiation therapy on the chest, or if they underwent emergency surgery or a redo surgery. Other exclusion criteria included non-centralized/midline sternotomy, the presence of a transverse fracture, cases where bilateral internal thoracic artery harvesting was performed, and cases requiring reoperation for bleeding.

### Sample Size Calculation

Sample size was calculated based on the primary outcome of sternal dehiscence. Anticipating a difference of 12% in the incidence of sternal dehiscence between the closure techniques based on previous studies ( *e.g.* , 1.35% *vs.* 12%)^[ 13 ]^, a minimum of 150 patients (50 per group) was required to achieve 80% power at a two-sided significance level of 5% (alpha). To account for potential dropouts, the total sample size was increased by 10% to 165 patients, with 55 patients allocated to each of the three study groups.

### Patients’ Randomization and Allocation

Randomization was achieved using computer-generated random sequences, which were concealed in sealed, opaque envelopes. Participants and data analysts were blinded to the group assignment. However, surgeons performing the closure could not be blinded due to the nature of the intervention. Patients were randomly allocated into three groups based on the method of sternal closure: (1) Group A received simple interrupted stainless-steel sutures method, (2) Group B received figure-of-eight stainless-steel sutures method, and (3) Group C received modified combined method of simple interrupted sutures, with the exception of one figure-of-eight suture placed specifically at the sternal angle.

### Patients’ Preparation and Sternal Procedures

Preoperatively, all patients underwent a comprehensive assessment, followed by an antiseptic shower with povidone-iodine, and chest hair was removed via clipping on the morning of surgery. Just before incision, the skin was disinfected with 10% povidone-iodine, and prophylactic antibiotics were administered 30 - 60 minutes before incision. Following the median sternotomy and the necessary cardiac procedure, sternal closure was performed using No. 7 stainless-steel wires, with patients randomly allocated to one of three groups: Group A, receiving the simple interrupted technique (six to eight wires); Group B, receiving the figure-of-eight technique (four complete wires); or Group C, receiving a modified combined method that used simple interrupted sutures with one stabilizing figure-of-eight wire placed at the sternal angle. The left internal mammary artery (LIMA) was harvested using a pedicled technique in all cases. Periosteal bleeders were managed with pinpoint cautery to avoid extensive devascularization of the sternal edges.

In all groups, wires were initially hand-twisted, followed by vertical traction and final tightening using a needle holder to ensure optimal sternal approximation before the muscular and cutaneous layers were closed. A unified analgesia protocol was implemented, and a chest belt was applied to support sternal stability. Patients were advised to avoid lateral sleeping positions or placing excessive pressure on the upper limbs during the recovery period.

### Study Outcomes

The primary outcome was sternal dehiscence (defined as the separation of sternal halves by > 2 mm within 30 postoperative days with clinical and radiographical confirmation). Secondary outcomes included sternal wound infections (either superficial or deep) classified per Centers for Disease Control and Prevention (2019) definitions^[ 14 ]^. In addition, postoperative pain (assessed using Numeric Rating Scale at 72 hours), sternal closure time, duration of mechanical ventilation, vasoactive inotropic score (VIS)^[ 15 ]^, 24-hour postoperative bleeding, and the lengths of intensive care unit (ICU) and total hospital stays.

### Statistical Analysis

Statistical analysis was conducted using Jamovi software (version 2.6.26). Continuous data were reported as mean ± standard deviation and compared across the three groups using one-way analysis of variance. Categorical data were expressed as frequencies and compared using Pearson’s Chi-squared test. The variables analyzed included baseline demographics, intraoperative details, and postoperative outcomes. Univariate logistic regression was used for the exploratory analysis of risk factors. The results of the regression analysis are reported as odds ratios (ORs) with corresponding 95% confidence intervals. A two-tailed *P* -value of < 0.05 was considered to indicate statistical significance.

## RESULTS

### Patient Demographics and Baseline Characteristics

A total of 165 patients were enrolled and randomized into three study groups (Group A, N = 55; Group B, N = 55; Group C, N = 55). There were no statistically significant differences observed among the three groups with respect to baseline characteristics, risk factors, or preoperative laboratory parameters ( Table 1 ). The total cohort consisted of 85 (51.5%) male and 80 (48.5%) female patients, with a mean age of 63.3 ± 4.6 years and a mean body mass index (BMI) of 29.6 ± 1.7 kg/m^2^. The prevalence of major risk factors, including diabetes mellitus (46.7%), hypertension (64.8%), and smoking (49.7%), was comparable across all three intervention groups ( *P* = 0.729, *P* = 0.374).

**Table 1.** Baseline characteristics, risk factors, and laboratory tests between study groups.

Variables		Group A (N = 55)	Group B (N = 55)	Group C (N = 55)	Total (N = 165)	*P* -value
Sex	Female	32 (58.2%)	22 (40%)	26 (47.3%)	80 (48.5%)	0.158
	Male	23 (41.8%)	33 (60%)	29 (52.7%)	85 (51.5%)	
Age (years)		62.3 (4.4)	63.2 (4.7)	64.4 (4.3)	63.3 (4.6)	0.051
BMI (kg/m^2^)		30.0 (1.8)	29.4 (1.5)	29.2 (1.6)	29.6 (1.7)	0.076
EF (%)		55.8 (7.2)	55.8 (7.4)	54.7 (8.4)	55.4 (7.7)	0.066
NYHA class	I	8 (14.5%)	5 (9.1%)	9 (16.4%)	22 (13.3%)	0.396
	II	21 (38.2%)	22 (40%)	17 (30.9%)	60 (36.4%)	
	III	23 (41.8%)	18 (32.7%)	22 (40.0%)	63 (38.2%)	
	IV	3 (5.5%)	10 (18.2%)	7 (12.7%)	20 (12.1%)	
DM	Yes	24 (43.6%)	25 (45.5%)	28 (50.9%)	77 (46.7%)	0.729
	No	31 (56.4%)	30 (54.5%)	27 (49.1%)	88 (53.3%)	
HTN	Yes	32 (58.2%)	39 (70.9%)	36 (65.5%)	107 (64.8%)	0.374
	No	23 (41.8%)	16 (29.1%)	19 (34.5%)	58 (35.2%)	
Smoking	Yes	25 (45.5%)	31 (56.4%)	26 (47.3%)	82 (49.7%)	0.472
	No	30 (54.5%)	24 (43.6%)	29 (52.7%)	83 (50.3%)	
COPD	Yes	6 (10.9%)	10 (18.2%)	8 (14.5%)	24 (14.5%)	0.557
	No	49 (89.1%)	45 (81.8%)	47 (85.5%)	141 (85.5%)	
CKD	Yes	5 (9%)	3 (5.4%)	6 (10.9%)	14 (8.4%)	0.303
	No	50 (91%)	52 (94.6%)	49 (80.1%)	151 (81.6%)	
Obesity	Yes	17 (30.9%)	27 (49.1%)	22 (40%)	66 (40%)	0.15
	No	38 (69.1%)	28 (50.9%)	33 (60.0%)	99 (60%)	
Hb (gm/dL)		14.1 (1.1)	13.3 (0.9)	12.9 (1.2)	12.1 (1.1)	0.278
Platelets		225.9 (29.2)	220.4 (31.0)	218.2 (31.4)	221.5 (30.5)	0.508
TLC		6.4 (0.9)	6.3 (0.9)	6.2 (0.9)	6.3 (0.9)	0.633
CRP		5.4 (1.9)	4.9 (1.6)	5.0 (1.8)	5.1 (1.8)	0.321
Albumin		3.8 (0.3)	3.7 (0.4)	3.6 (0.4)	3.7 (0.4)	0.158
HbA1C		7.6 (1.7)	7.4 (1.8)	7.3 (1.8)	7.4 (1.8)	0.667

Data was presented as mean (standard deviation) or n (%)

BMI=body mass index; CKD=chronic kidney disease; COPD=chronic obstructive pulmonary disease; CRP=C-reactive protein; DM=diabetes mellitus; EF=ejection fraction; Hb=hemoglobin; HTN=hypertension; NYHA=New York Heart Association; TLC=total leukocyte count

### Surgical Procedures and Intraoperative Parameters

Analysis of the surgical procedures and intraoperative metrics also demonstrated homogeneity across the three study groups ( Table 2 ). There were no statistically significant differences observed in the type of surgery performed (coronary artery bypass grafting was the most frequent at 40.6% of total cases, *P* = 0.529), pump strategy (on-pump 77%, *P* = 0.787), or the rate of LIMA usage ( *P* = 0.903). Furthermore, key intraoperative times, including cardiopulmonary bypass (CPB) time (94.0 ± 18.0 min, *P* = 0.373), aortic cross-clamping time (65.2 ± 13.9 min, *P* = 0.488), and total operative time (289.0 ± 45.7 min, *P* = 0.696), were similar across Groups A, B, and C. Sternal closure time showed a trend toward difference between groups (14.1 ± 2 min, *P* = 0.052), although it did not reach the conventional threshold for statistical significance.

**Table 2.** Comparison of surgical procedures and intraoperative parameters across study groups.

Variables		Group A (N = 55)	Group B (N = 55)	Group C (N = 55)	Total (N = 165)	*P* -value
Surgery	CABG	25 (45.5%)	22 (40%)	20 (36.4%)	67 (40.6%)	0.529
	MVR	7 (12.7%)	8 (14.5%)	7 (12.7%)	22 (13.3%)	
	AVR	5 (9.1%)	9 (16.4%)	13 (23.6%)	27 (16.4%)	
	DVR	4 (7.3%)	4 (7.3%)	4 (7.3%)	12 (7.3%)	
	CABG + MVR	6 (10.9%)	1 (1.8%)	2 (3.6%)	9 (5.5%)	
	ASD	3 (5.5%)	5 (9.1%)	1 (1.8%)	9 (5.5%)	
	CABG + AVR	3 (5.5%)	1 (1.8%)	3 (5.5%)	7 (4.2%)	
	SAM	1 (1.8%)	3 (5.5%)	4 (7.3%)	8 (4.8%)	
	TVR	1 (1.8%)	2 (3.6%)	1 (1.8%)	4 (2.4%)	
On/off pump	On	41 (74.5%)	42 (76.4%)	44 (80%)	127(77%)	0.787
	Off	14 (25.5%)	13 (23.6%)	11 (20%)	38 (23.0%)	
LIMA usage	Yes	34 (61.8%)	24 (43.6%)	25 (45.4%)	100 (60.6%)	0.903
	No	21 (38.2%)	31 (56.4%)	30 (54.6%)	65 (39.4%)	
CPB time (min)		97.2 (18)	93 (18)	91.9 (18.2)	94 (18)	0.373
Aortic cross-clamping time (min.)		66.9 (14.2)	63.7 (13.6)	65 (13.8)	65.2 (13.9)	0.488
Sternal closure time (min.)		15.2 (1.8)	13.0 (1.7)	14.0 (1.8)	14.1 (2)	0.052
Operative time (min.)		292.7 (45.8)	289 (46.3)	285.3 (45.6)	289 (45.7)	0.696

Data was presented as mean (standard deviation) or n (%)

ASD=atrial septal defect; AVR=aortic valve replacement; CABG=coronary artery bypass grafting; CPB=cardiopulmonary bypass; DVR=double valve replacement; LIMA=left internal mammary artery; MVR=mitral valve replacement; SAM=subaortic membrane; TVR=tricuspid valve replacement

### Postoperative Clinical Outcomes and Complications

Postoperative outcomes and complication rates were comparable across the three study groups, with no statistically significant differences found in any of the analyzed variables ( Table 3 ). Mean mechanical ventilation duration was 7.9 ± 3.5 hours ( *P* = 0.301), and mean hospital stay was 7.0 ± 3.4 days ( *P* = 0.451). The overall incidence of SWCs was 6.1% for sternal dehiscence, 7.9% for superficial, and 3.6% for DSWI. The rates of these complications, as well as measures like postoperative bleeding and VIS, were not statistically different between the intervention groups.

**Table 3.** Postoperative clinical outcomes and complications across study groups.

Variables		Group A (N = 55)	Group B (N = 55)	Group C (N = 55)	Total (N = 165)	*P* -value
MV duration (hours)		8.5 (3.5)	7.4 (3.5)	7.8 (3.5)	7.9 (3.5)	0.301
Bleeding (ml)		480.2 (123)	470.5 (122)	460.4 (122)	470 (121.9)	0.698
Vasoactive inotropic score		5.5 (4)	5.1 (3.7)	4.7 (3.4)	5.1 (3.7)	0.214
Numeric rating scale		4.5 (2.1)	4.2 (2)	3.9 (2)	4.2 (2.1)	0.192
Hospital stays (days)		7.2 (3.6)	7 (3.5)	6.8 (3.2)	7 (3.4)	0.451
Sternal dehiscence	Yes	5 (9.1%)	3 (5.5%)	2 (3.6%)	10 (6.1%)	0.361
	No	50 (90.9%)	52 (94.5%)	53 (96.4%)	155 (93.9%)	
SSWI	Yes	6 (10.9%)	4 (7.3%)	3 (5.5%)	13 (7.9%)	0.361
	No	49 (89.1%)	51 (92.7%)	52 (94.5%)	152 (92.1%)	
DSWI	Yes	3 (5.5%)	2 (3.6%)	1 (1.8%)	6 (3.6%)	0.166
	No	52 (94.5%)	53 (96.4%)	54 (98.2%)	159 (96.4%)	

Data was presented as mean (standard deviation) or n (%)

DSWI=deep sternal wound infection; MV=mechanical ventilation; SSWI=superficial sternal wound infection

### Potential Risk Factors Associated with Sternal Dehiscence

Binomial logistic regression identified several significant factors associated with sternal dehiscence ( Table 4 ). Patient-related factors included increasing age (OR: 1.03, *P* = 0.049), obesity (BMI ≥ 30; OR: 2.48, *P* = 0.042), diabetes mellitus (OR: 3.05, *P* = 0.014), smoking (OR: 1.89, *P* = 0.024), chronic obstructive pulmonary disease (COPD) (OR: 2.75, *P* = 0.042), and elevated C-reactive protein (CRP) (OR: 2.92, *P* = 0.026). Operative factors significantly associated with dehiscence were prolonged operative time (OR: 2.35, *P* = 0.047), extended CPB time (OR: 2.60, *P* = 0.038), and longer ICU stay (OR: 2.80, *P* = 0.032).

**Table 4.** Binomial logistic regression analysis for significant risk factors associated with sternal dehiscence following cardiac surgery.

Factors	Odds ratio	95% CI	*P* -value
		(lower – upper)	
Age (years)	1.03	1.00 – 1.07	0.049
BMI ≥ 30	2.48	1.03 – 5.97	0.042
Diabetes mellitus (yes *vs.* no)	3.05	1.25 – 7.43	0.014
Smoking (yes *vs.* no)	1.89	0.84 – 4.23	0.024
COPD (yes *vs.* no)	2.75	0.95 – 7.96	0.042
Serum albumin < 3.5 g/dL	3.88	1.45 – 10.41	0.007
CRP (mg/L)	2.92	1.13 – 7.55	0.026
Operative time (min)	2.35	1.01 – 5.50	0.047
CPB time (min)	2.60	1.06 – 6.39	0.038
ICU stay	2.80	1.09 – 7.21	0.032

BMI=body mass index; CI=confidence interval; COPD=chronic obstructive pulmonary disease; CPB=cardiopulmonary bypass; CRP=C-reactive protein; ICU=intensive care unit

### Potential Risk Factors Associated with Superficial Sternal Wound Infection

Similar to dehiscence, multiple factors were identified as potential factors associated with for superficial sternal wound infection ( Table 5 ). Patient characteristics associated with increased risk included increasing age (OR: 1.04, *P* = 0.015), obesity (BMI ≥ 30; OR: 2.85, *P* = 0.015), diabetes mellitus (OR: 3.41, *P* = 0.003), smoking (OR: 2.06, *P* = 0.043), COPD (OR: 2.94, *P* = 0.048), and elevated CRP (OR: 3.01, *P* = 0.014). Operative factors included prolonged operative time (OR: 2.73, *P* = 0.027), extended CPB time (OR: 2.89, *P* = 0.019), and longer ICU stay (OR: 2.58, *P* = 0.028).

**Table 5.** Binomial logistic regression analysis for significant risk factors associated with superficial sternal wound infection following cardiac surgery.

Factors	Odds ratio	95% CI	*P* -value
		(lower – upper)	
Age (years)	1.04	1.01 – 1.08	0.015
BMI ≥ 30	2.85	1.22 – 6.64	0.015
Diabetes mellitus	3.41	1.51 – 7.71	0.003
Smoking	2.06	0.96 – 4.42	0.043
COPD	2.94	1.01 – 8.54	0.048
Serum albumin < 3.5 g/dL	4.22	1.70 – 10.47	0.002
CRP	3.01	1.25 – 7.27	0.014
Operative time	2.73	1.12 – 6.67	0.027
CPB time	2.89	1.19 – 7.04	0.019
ICU Stay	2.58	1.11 – 6.03	0.028

BMI=body mass index; CI=confidence interval; COPD=chronic obstructive pulmonary disease; CPB=cardiopulmonary bypass; CRP=C-reactive protein; ICU=intensive care unit

### Potential Risk Factors Associated with Deep Sternal Wound Infection

The logistic regression analysis for DSWI identified a distinct set of significant risk factors ( Table 6 ). These included advancing age (OR: 1.04, *P* = 0.014), increasing BMI (OR: 1.10, *P* = 0.008), diabetes mellitus (OR: 2.90, *P* = 0.007), smoking (OR: 2.35, *P* = 0.030), chronic kidney disease (CKD) (OR: 3.10, *P* = 0.022), and elevated HbA1C (OR: 1.29, *P* = 0.034). Prolonged CPB time was the only intraoperative factor that remained statistically significant (OR: 1.03, *P* = 0.042).

**Table 6.** Binomial logistic regression analysis for significant risk factors associated with deep sternal wound infection following cardiac surgery.

Factors	Odds ratio	95% CI	*P* -value
		(lower – upper)	
Age (years)	1.04	1.01 – 1.08	0.014
BMI	1.10	1.02 – 1.18	0.008
Diabetes mellitus (yes/no)	2.90	1.34 – 6.26	0.007
Smoking (yes/no)	2.35	1.08 – 5.09	0.03
CKD (yes/no)	3.10	1.18 – 8.13	0.022
HbA1C	1.29	1.02 – 1.62	0.034
CBP time	1.03	1.00 – 1.06	0.042

BMI=body mass index; CI=confidence interval; CKD=chronic kidney disease; CPB=cardiopulmonary bypass

## DISCUSSION

This randomized controlled study was designed to compare the efficacy of three distinct steel-wire closure techniques in a cohort of 165 adult cardiac surgery patients, focusing on early postoperative stability, complication rates, and identifying independent risk factors. Our findings demonstrate that all three sternal closure techniques resulted in comparable intraoperative parameters and immediate postoperative recovery.

Specifically, no statistically significant differences were observed across the groups in the type of surgery performed, on/off pump strategy, LIMA usage, CPB time, aortic cross-clamping time, or total operative time. Our results align with previous studies which reported no significant differences in intraoperative variables when comparing various sternal closure methods^[ 16 - 18 ]^. The non-significant difference in sternal closure time was consistent with the observation by Mourad et al.^[ 16 ]^ that specific techniques may be marginally longer, though not clinically or statistically distinct in this context. Similarly, the postoperative recovery metrics were comparable across all three groups. We found no significant differences in mechanical ventilation duration, bleeding, VIS, pain scores, ICU stay, or total hospital stay. These results are consistent with a study conducted by Duzgun et al.^[ 19 ]^ who reported no statistically significant differences in these early recovery parameters between different sternal closure methods, suggesting that the choice among the three tested wire techniques does not substantially impact short-term clinical recovery trajectory.

Although a downward trend in complications was numerically suggested in some groups, the differences remained comparable across all methods. This finding is in line with multiple comparative studies which demonstrated that polyethylene suture tapes, sternal cables, or bioresorbable plates did not yield significantly lower complication rates compared to traditional wire techniques^[ 20 , 21 ]^. The absence of a significant difference suggests that the biomechanical stability provided by these three wire-based methods is equivalent in preventing early superficial SWCs.

Conversely, Mourad et al.^[ 16 ]^ did report a significant difference in dehiscence rates between their groups which contrasts with our findings. However, they found both superficial and deep SWC rates to be statistically non-significant, similar to our results. This discrepancy may be attributable to differences in patient selection, specific closure protocols, or baseline risk profiles between the study populations.

Despite the lack of difference between the closure techniques, our binomial logistic regression analysis successfully identified multiple significant patient- and procedure-related risk factors for superficial SWCs. Significant potential risk factors included old age, obesity, diabetes, smoking, CKD, COPD, elevated CRP and HbA1C, prolonged CPB time, extended operative time, and longer ICU stay. These findings are strongly supported by extensive literature identifying major risk factors for superficial SWCs. Additionally, patient-related risk factors such as obesity, diabetes, and COPD were confirmed as independent predictors by Duzgun et al.^[ 19 ]^, Arribas-Leal et al.^[ 22 ]^, and Meszaros et al.^[ 23 ]^.

Who noted the significance of low serum albumin. Furthermore, Pan et al.^[ 24 ]^ confirmed the risk posed by age, BMI, and diabetes for deep SWCs. Procedural factors including prolonged CPB time, operative time, and ICU stay, are recognized markers of surgical complexity and patient fragility. Their inclusion as specific risk factors in our model reinforces the understanding that minimizing these variables is crucial for reducing postoperative risk.

### Limitations

This study has some limitations that should be considered. The single-center design may restrict the generalizability of the results, particularly to institutions with different patient populations or surgical protocols. Furthermore, the absence of long-term follow-up precludes assessment of late complications and the durability of the sternal closures. Additionally, the secondary analysis of risk factors for SWCs was exploratory in nature. Given the relatively low number of outcome events (10 sternal dehiscences, 13 superficial infections, six deep infections), the logistic regression models were at risk of overfitting. Therefore, the identified associations should be interpreted as hypothesis-generating rather than confirmatory, and they require validation in larger, adequately powered prospective cohorts or registry-based studies.

## CONCLUSION

In this RCT, the three investigated steel-wire sternal closure techniques (simple interrupted, figure-of-eight, and a modified combined technique) demonstrated comparable early postoperative stability and resulted in statistically similar rates of SWCs. While several patient- and procedure-related factors were identified as potential risk factors for these complications, these findings should be interpreted with caution due to the exploratory nature of the analysis and the limited number of events. The choice among these wire techniques can be based on surgeon preference and experience without expecting a significant difference in early outcomes.

## Data Availability

The authors declare that the data will be available upon reasonable request to the authors.
